# Retrospective review of safety and efficacy of programmed cell death-1 inhibitors in refractory high grade gliomas

**DOI:** 10.1186/s40425-017-0302-x

**Published:** 2017-12-19

**Authors:** Samantha N. Reiss, Prakirthi Yerram, Lisa Modelevsky, Christian Grommes

**Affiliations:** 1 Department of Pharmacy, Memorial Sloan Kettering Cancer Center, 1275 York Ave, New York, NY 10065 USA; 2 Department of Neurology, Memorial Sloan Kettering Cancer Center, 1275 York Ave, New York, NY 10065 USA

**Keywords:** Glioblastoma, Immune checkpoint, High-grade glioma, PD-1, PD-L1, Pembrolizumab

## Abstract

**Background:**

Programmed cell death ligand-1 (PD-L1) expression has been reported in up to 61% of high grade gliomas (HGG). The purpose of this study was to describe safety and efficacy of PD-1 inhibition in patients with refractory HGGs.

**Methods:**

This Institutional Review Board approved single center retrospective study included adult patients with pathologically confirmed HGG who received a PD-1 inhibitor from 9/2014–10/2016 outside of a clinical trial at Memorial Sloan Kettering Cancer Center.

**Results:**

Twenty five HGG patients received pembrolizumab as part of a compassionate use program. Median age was 50 years (range 30–72); 44% were men; 13 had glioblastoma (52%), 7 anaplastic astrocytoma (28%), 2 anaplastic oligodendroglioma (8%), 2 unspecified HGG (8%), and 1 gliosarcoma (4%). Median prior lines of treatments were 4 (range 1–9). Nineteen (76%) previously failed bevacizumab. Median KPS was 80 (range 50–100). Concurrent treatment included bevacizumab in 17 (68%) or bevacizumab and temozolomide in 2 (8%) patients. Median number of doses administered was 3 (range 1–14). Outcomes were assessed in 24 patients. PD-1 inhibitor related adverse events included LFT elevations, hypothyroidism, diarrhea, myalgias/arthralgias, and rash. Best radiographic response was partial response (*n* = 2), stable disease (*n* = 5), and progressive disease (*n* = 17). Median progression free survival (PFS) was 1.4 months (range 0.2–9.4) and median overall survival (OS) was 4 months (range 0.5–13.8). Three-month PFS was 12% and 6-month OS was 28%.

**Conclusion:**

While response rates are low, a few patients had a prolonged PFS. Pembrolizumab was tolerated with few serious toxicities, even in patients receiving concomitant therapy.

## Background

High grade malignant gliomas, including anaplastic oligodendrogliomas, anaplastic astrocytoma (grade III) and glioblastomas (grade IV), are the most common primary malignant brain tumors diagnosed in adults [1]. Despite advancements in understanding the underlying pathogenesis, overall survival remains limited with a median survival for glioblastoma, the most aggressive high grade glioma (HGG), between 16 and 19 months [1]. Upfront therapy for glioblastoma consists of maximal safe resection followed by radiation with concurrent temozolomide and adjuvant temozolomide [2]. Median survival for patients with recurrent grade III and grade IV tumors is 39 and 30 weeks, respectively [3]. Progression free survival at 26 weeks is 28% for grade III tumors and 16% for grade IV tumors. Non-surgical treatment options for recurrent or progressive high grade gliomas are limited. FDA approved treatment options for recurrent glioblastoma include an anti-vascular endothelial growth factor (VEGF) agent, bevacizumab, and low-intensity alternating electric fields (TTFields); neither treatment has been shown to significantly improve overall survival [4–6]. Other treatment options include conventional chemotherapy such as temozolomide in different dosing schedules, carboplatin, irinotecan, and nitrosoureas [7].

Checkpoint inhibitors have advanced treatment for metastatic melanoma, non-small cell lung cancer, renal cell carcinoma, Non-Hodgkin Lymphoma and other malignancies [8, 9]. For patients diagnosed with non-small cell lung cancer, the level of programmed cell death ligand-1 (PD-L1) expression has been associated with improved outcomes to PD-1 inhibitors [8, 10, 11]. The presence of tumor infiltrating lymphocytes and PD-L1 expression has been reported in up to 61% of high grade gliomas and therefore this checkpoint is a viable target for treatment [12, 13]. PD-1 inhibitors block the interaction between PD-L1 and its receptor thereby overcoming T-cell inhibition and promoting an immune response against the tumor. Developing effective treatment options for malignant high grade gliomas has proven difficult due to the inability of many medications to cross the blood brain barrier. Data evaluating the penetration of checkpoint inhibitors across the blood brain barrier is limited. However, the activity of immunotherapy for brain metastasis from melanoma and lung cancer has been reported and is promising [14]. Additionally, there have been case reports of prolonged response after checkpoint inhibitors in patients with glioblastoma [15, 16]. Currently, there are an abundance of clinical trials evaluating checkpoint inhibitors of patients with glioblastoma. Unfortunately, many patients with high grade gliomas are excluded due to previous treatments, performance status, or tumor histology [12, 17, 18]. At our institution, many patients with high grade gliomas that do not qualify for clinical trial receive off label checkpoint inhibitors. The purpose of this retrospective study is to describe efficacy and safety of PD-1 inhibitors in patients with refractory malignant high grade gliomas.

## Methods

### Study design

This was an Institutional Review Board approved single-center observational retrospective study performed at Memorial Sloan Kettering Cancer Center evaluating patients with pathology confirmed high grade malignant glioma who received a PD-1 inhibitor outside of a clinical trial. Patients were identified through the pharmacy database and electronic medical records. Inclusion criteria consisted of patients who were 18 years of age or older and had received a PD-1 inhibitor between September 2014 and October 2016. Patients were excluded if they received a PD-1 inhibitor as part of a clinical trial.

### Endpoints and assessments

The primary objective of this study was to describe overall response rate (ORR) on contrast enhanced MRI. Secondary objectives included characterizing toxicities according to the Common Terminology Criteria for Adverse Events (CTCAE) version 4.03 as well as describing progression free survival (PFS) and overall survival (OS). Frequencies and percentages were used to describe categorical variables and medians and ranges were used to describe continuous variables. Kaplan-Meier methods were used to visualize PFS and OS; patients were censored at the last follow up date if an event did not occur.

## Results

### Patient characteristics

Twenty-nine neuro-oncology patients received a PD-1 inhibitor between September 2014 and October 2016. Four patients were excluded; 3 patients received previous checkpoint inhibitor therapy as part of a clinical trial and 1 patient did not have a high grade glioma. Baseline characteristics are described in Table 1. The median age was 49 years (range: 30–72 years), 11 patients were male (44%), and the majority of patients were Caucasian (88%). All patients received pembrolizumab as PD-1 inhibitor for treatment of HGG through a compassionate use program. Thirteen patients had pathology confirmed glioblastoma (52%), 7 anaplastic astrocytoma (28%), 2 anaplastic oligodendroglioma (8%), 2 unspecified HGG (8%), and 1 gliosarcoma (4%). Four patients (16%) were MGMT methylated, 12 (48%) were MGMT unmethylated and 9 (36%) were unknown. Ten patients (40%) had tumors that harbored an IDH1 mutation, 9 (36%) were IDH1 wild type, and 6 (24%) were unknown. Median mutational load was 7 with a range of 3–58 (Table 2). None of the patients were considered to have a hypermutator phenotype, defined as 100 or more mutations, by MSK-Impact.^16^ Patients were heavily pretreated, receiving a median of 4 prior lines of therapy (range 1–9) and 19 patients (76%) previously progressed on bevacizumab treatment. Median KPS at initiation of pembrolizumab was 80 (range 50–100). Concurrent treatment with pembrolizumab included bevacizumab in 17 (68%) or bevacizumab and temozolomide in 2 (8%) patients. Out of the 19 patients who previously failed bevacizumab, 17 continued on bevacizumab with pembrolizumab therapy. Of the six patients who did not previously receive bevacizumab therapy, two were started on bevacizumab in combination with pembrolizumab. Median number of doses of pembrolizumab administered was 3 (range 1–14). Fourteen patients (56%) were on dexamethasone during their first treatment dose and 19 patients (79%) received dexamethasone at some point during the course of treatment with pembrolizumab. Out of the 105 total doses of pembrolizumab administered, 34 doses (32%) were administered with concomitant dexamethasone for treatment of disease related neurologic symptoms.

**Table 1:** Baseline Characteristics

Characteristic	All patients (*n* = 25)
Age, year (range)	49 (30–72)
Gender: male, no. (%)	11 (44)
Race	
Caucasian, no. (%)	22 (88)
Asian, no. (%)	1 (4)
Black, no. (%)	0 (0)
Latino/Hispanic, no. (%)	1 (4)
Other, no. (%)	1 (4)
Diagnosis	
Glioblastoma, no. (%)	13 (52)
Anaplastic astrocytoma, no. (%)	7 (28)
Anaplastic oligodendroglioma, no. (%)	2 (8)
Unspecified high grade glioma, no. (%)	2 (8)
Gliosarcoma, no. (%)	1 (4)
Performance status, KPS (range)	80 (50–100)
Number of prior therapies, median (range)	4 (1–9)
Previously received bevacizumab, no. (%)	19 (76)
MGMT status	
Methylated, no. (%)	4 (16)
Unmethylated, no. (%)	12 (48)
Unknown, no. (%)	9 (36)
IDH1 Status	
IDH1 Mutated, no. (%)	10 (40)
IDH1 Wild Type, no. (%)	9 (36)
Unknown, no. (%)	6 (24)
Number of mutations by MSK-Impact, median (range)	7 (3–58)
PD-1 inhibitor	
Pembrolizumab, no. (%)	25 (100)
Number of doses administered, median (range)	3 (1–14)
Concomitant therapy	
Pembrolizumab monotherapy, no. (%)	6 (24)
Bevacizumab, no. (%)	17 (68)
Cytotoxic chemotherapy + bevacizumab, no. (%)	2 (8)
Receiving dexamethasone at time of first dose, no. (%)	14 (56%)

**Table 2:** Patient Characteristics, Response and Steroid Dose

Pt #	Grade	OR	KPS	# of cycles of pembro	MGMT Status	IDH Status	1p/19q	ML	Steroids at initiation	Steroid dose at initiation (in prednisone equivalence)			# of cycles with steroids	Con Bev	Prev Bev
										0	<20	≥20			
1	III	PR	90	4	unmethylated	WT	N/A	6	N	X			2	Y	Y
2	IV	PR	80	4	unmethylated	N/A	N/A	6	Y			X	1	Y	N
3	III	SD	90	14	methylated	WT	intact	6	N	X			0	N	N
4	III	SD	70	10	unmethylated	MUT	N/A	3	Y			X	1	Y	Y
5	IV	SD	90	14	unmethylated	WT	N/A	12	N	X			5	Y	Y
6	IV	SD	100	4	methylated	N/A	N/A	13	N	X			0	N	Y
7	IV	SD	100	1	N/A	WT	N/A	N/A	Y			X	1	Y	Y
8	III	PD	90	6	N/A	MUT	co-del	5	Y		X		1	N	N
9	III	PD	60	5	N/A	MUT	co-del	58	Y		X		0	Y	Y
10	III	PD	60	3	unmethylated	MUT	N/A	7	N	X			2	N	N
11	III	PD	70	3	unmethylated	WT	N/A	5	Y		X		3	Y	Y
12	III	PD	90	2	unmethylated	WT	intact	15	Y		X		2	Y	N
13	III	PD	90	2	unmethylated	MUT	N/A	7	Y			X	2	Y	Y
14	III	PD	N/A	1	methylated	MUT	N/A	5	N	X			0	N	Y
15	IV	PD	90	5	methylated	MUT	N/A	11	Y			X	2	N	N
16	IV	PD	60	5	unmethylated	WT	intact	10	Y			X	2	Y	Y
17	IV	PD	50	4	N/A	N/A	N/A	N/A	N	X			0	Y	Y
18	IV	PD	90	3	N/A	MUT	N/A	9	Y			X	3	Y	Y
19	IV	PD	90	3	unmethylated	N/A	N/A	4	Y			X	1	Y	Y
20	IV	PD	90	3	N/A	N/A	N/A	N/A	N	X			1	Y	Y
21	IV	PD	70	2	unmethylated	WT	N/A	13	Y		X		2	Y	Y
22	IV	PD	80	2	unmethylated	MUT	N/A	5	N	X			0	Y	Y
23	IV	PD	80	2	N/A	WT	N/A	N/A	N	X			1	Y	Y
24	N/A	PD	70	2	N/A	N/A	N/A	N/A	N	X			1	Y	Y
25	IV	N/A	60	1	N/A	MUT	intact	19	Y			X	1	Y	Y

Abbreviations: Pt: Patient; OR: Objective response; CR: Complete response; PR: Partial response; SD: Stable disease; PD: Progressive disease; KPS: Karnofsky performance score; Pembro: pembrolizumab; N/A: not applicable or unknown; MGMT methylated: methylated; MGMT unmethylated: unmethylated; IDH mutant: MUT; IDH wild type: WT; 1p19q intact: intact; 1p19q codeleted: Co-del; ML: mutational load by MSK impact; Y: yes; N: no; X: indicates steroid dose at initiation; Con Bev: Concomitant bevacizumab; Prev Bev: previously progressed on bevacizumab treatment

### Efficacy

**Fig. 1:**
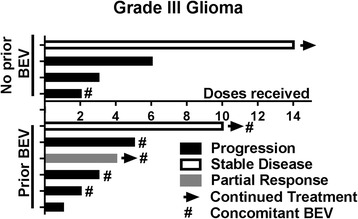
Best Radiographic Response in Grade III Glioma Patients. BEV = bevacizumab. The X axis represents the number of doses of pembrolizumab that was received. The color represents the best radiographic response each patient had. 3 patients continue on pembrolizumab at the end of data collection. 6 patients previously progressed on bevacizumab; of those patients 5 continued bevacizumab with pembrolizumab. 4 patients never received bevacizumab, of those 1 started on bevacizumab with pembrolziumab. One patient had a partial response; 2 had stable disease; and the rest had progressive disease

**Fig. 2:**
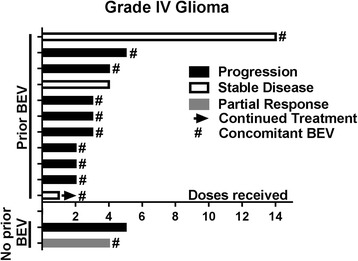
Best Radiographic Response in Grade IV Glioma Patients. BEV = bevacizumab. The X axis represents the number of doses of pembrolizumab that was received by each patient. The color represents the best radiographic response each patient had. One patient continue on pembrolizumab at the end of data collection. Eleven patients previously progressed on bevacizumab; of those patients 10 continued bevacizumab with pembrolizumab. 2 patients never received bevacizumab and, of those, one started on bevacizumab with pembrolziumab. One patient had a partial response; 3 had stable disease; and the rest had progressive disease

**Fig. 3:**
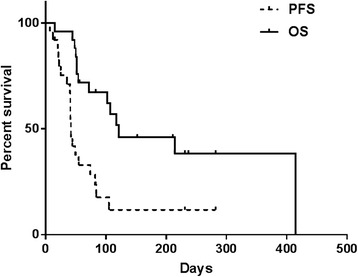
Overall Survival and Progression Free Survival. Median progression free survival (PFS) was 1.4 months (range 0.2–9.4) and median overall survival (OS) was 4 months (range 0.5–13.8). Six month PFS was 12% and 6 month OS was 28%. Two patients had stable disease greater than 200 days

Treatment response and toxicity was evaluable in 24 patients. One patient was excluded from evaluation of response and toxicity because they transitioned to hospice less than one week after their first and only dose of pembrolizumab; therefore, imaging and toxicity data is not available. This patient was included in survival analysis. Best radiographic response was partial response (*n* = 2, 8%), stable disease (*n* = 5, 21%), and progressive disease (*n* = 17, 71%) (Table 3). Both of the patients with a partial response received concomitant bevacizumab, and one patient was bevacizumab-naïve. These two patients received pembrolizumab plus bevacizumab in the second and third line setting for treatment of glioblastoma and anaplastic astrocytoma, respectively. Both patients received dexamethasone for management of disease related symptoms, one at initiation of pembrolizumab treatment. Duration of therapy, best radiographic response, previous bevacizumab, and concomitant bevacizumab can be visualized in Figs. 1 and 2. Two patients had stable disease greater than 200 days. One of these patients received bevacizumab plus pembrolizumab after failing 9 prior treatments including bevacizumab containing regimens. The other patient received pembrolizumab monotherapy after failing 2 prior lines of therapy. The first patient was on dexamethasone only during their first dose of pembrolizumab. The second patient did not receive dexamethasone during treatment with pembrolizumab. Of note, 7 of the 18 patients without a clinical response did not require steroids at treatment initiation. The median mutation load was 6 in patients with partial response and stable disease compared to 7 in those who did not respond. Median progression free survival (PFS) was 1.4 months (range 0.2–9.4) and median overall survival (OS) was 4 months (range 0.5–13.8) (Fig. 3). Six month PFS was 12% and 6 month OS was 28%.

**Table 3:** Clinical Response

Characteristic	All evaluable patients (*n* = 24)
Best radiographic response	
Complete response (CR), no. (%)	0 (0)
Partial response (PR), no. (%)	2 (8)
Stable disease (SD), no. (%)	5 (21)
Progressive disease (PD), no. (%)	17 (71)
Median PFS, days (range)	42 (7–282)
Median OS, days (range)	121 (15–415)

### Toxicity

All toxicities are listed in Table 4. The most common adverse events reported were fatigue (grade 3–4: 4%; grade 1–2: 75%), headache (grade 3–4: 4%; grade 1–2: 43%), nausea (grade 3–4: 4%; grade 1–2: 37.5%), diarrhea (grade 3–4: 0%; grade 1–2: 17%), seizures (grade 3–4: 4%; grade 1–2: 17%), vomiting (grade 3–4: 4%; grade 1–2: 17%), myalgias/arthralgia (grade 3–4: 0%; grade 1–2: 13%), and rash (grade 3–4: 0%; grade 1–2: 8%). The most common laboratory abnormalities recorded were hyperglycemia (grade 1–2: 79%), thrombocytopenia (grade 1–2: 50%), leukopenia (grade 1–2: 37.5%), ALT elevations (grade 1–2: 33%), AST elevations (grade 1–2: 29%), bilirubin elevations (grade 1–2: 21%), neutropenia (grade 1–2: 21%), and hypothyroidism (grade 1–2: 17%). Additionally, 74% of patients (*n* = 14) who experienced hyperglycemia were receiving dexamethasone. One patient with a history of epilepsy was admitted for a grade 3 seizure. The second patient who experienced grade 3 adverse events, specifically nausea, vomiting, and headache, was admitted for symptoms of increased intracranial pressure due to pathology confirmed recurrent glioblastoma. Lastly, one patient experienced grade 4 cerebral edema requiring emergent surgery 7 days after their first and only dose of pembrolizumab. Pathology confirmed edema was due to rapid tumor progression. No patients discontinued pembrolizumab due to toxicity.

**Table 4:** Adverse events - incidence and grading according to CTCAE v 4.03

Toxicity	Overall incidence, no. (%)	Grade 1 and 2, no. (%)	Grade 3 and 4, no. (%)
Hyperglycemia	19 (79)	19 (79)	
Fatigue	19 (79)	18 (75)	1 (4)
Thrombocytopenia	12 (50)	12 (50)	
Headache	11 (46)	10 (43)	1 (4)
Nausea	10 (42)	9 (38)	1 (4)
Leukopenia	9 (38)	9 (38)	
ALT elevations	8 (33)	8 (33)	
AST elevations	7 (29)	7 (29)	
Bilirubin elevations	5 (21)	5 (21)	
Neutropenia	5 (21)	5 (21)	
Anemia	5 (21)	4 (17)	1 (4)
Seizures	5 (21)	4 (17)	1 (4)
Vomiting	5 (21)	4 (17)	1 (4)
Thyroid toxicity	4 (17)	4 (17)	
Diarrhea	4 (17)	4 (17)	
Myalgias/Arthralgias	3 (13)	3 (13)	
Rash	2 (8)	2 (8)	
Pyrexia	2 (8)	2 (8)	
Lipase	1 (4)	1 (4)	
Amylase	1 (4)	1 (4)	
Mucositis	1 (4)	1 (4)	

## Discussion

Our study demonstrated that heavily pretreated patients with malignant high grade gliomas have low response rates to pembrolizumab. To our knowledge, this is the first study to investigate PD-1 inhibition in grade III gliomas. Garber and colleagues found that PD-L1 expression was only present on grade IV gliomas, where as it was not present in the 33 anaplastic astrocytomas or 9 oligodendrogliomas. [19] There is no current data correlating PD-L1 expression and clinical outcomes outside of pembrolizumab use in non-small cell lung cancer. In our grade III glioma cohort, 1 patient had a partial response to pembrolizumab and 2 patients had prolonged progression free survival with pembrolizumab.

Pembrolizumab monotherapy for recurrent glioblastoma was studied in the KEYNOTE-028 trial. [20] Patients were included if they were diagnosed with glioblastoma having PD-L1 expression ≥1%, bevacizumab naïve, and unable to receive standard treatment. Median PFS and OS were reported as 2.8 months and 14.4 months, respectively. CheckMate-143 compared nivolumab monotherapy to bevacizumab monotherapy in glioblastoma in patients with first recurrence. Median OS was 9.8 months with nivolumab and 10 months with bevacizumab, PFS was 1.5 months with nivolumab and 3.5 months with bevacizumab, demonstrating no improvement in overall survival. [21] We observed a shorter PFS and OS most likely because patients that failed bevacizumab were also included.

Pembrolizumab was well tolerated in our cohort; toxicities were similar compared to those reported with other malignancies. [8, 9] Very few serious adverse events occurred during treatment. Serious adverse events, cerebral edema, seizures and headaches could be related to disease progression or checkpoint inhibition.

Our study had several limitations. Firstly, it was a retrospective study with a small sample size. Second, many patients received pembrolizumab in combination with other treatment modalities such as bevacizumab, making it difficult to evaluate the effectiveness of pembrolizumab monotherapy in high grade glioma patients. Additionally, we included patients with both WHO grade III and IV gliomas, making it difficult to compare these results to published data that includes only glioblastoma patients. Many of our patients were excluded from participation in clinical trials for checkpoint inhibitors due to their WHO grade, previous treatment with bevacizumab, and poor KPS. This patient population differs from previously reported clinical observations using checkpoint inhibitors as it includes grade III and IV gliomas. The observed response rate and survival data might be biased due to the poor prognostics factors in our population (heavily pretreated, bevacizumab-resistance, low KPS performance status). However, these patients are frequently encountered in the clinical setting with little literature to guide treatment decisions.

We also did not account for baseline abnormalities and due to the retrospective nature of this study were unable to differentiate between treatment related toxicity and disease related adverse events. Lastly, we did not assess PD-L1 expression to correlate clinical response to PD-L1 status. Pembrolizumab requires further studies to confirm a benefit for patients with refractory high grade glioma as monotherapy or in combination with chemotherapy or bevacizumab.

## Conclusions

Patients with pathology confirmed refractory high grade gliomas have low response rates to pembrolizumab. However, a small number of patients have a prolonged progression free survival. Pembrolizumab was tolerated with few serious adverse events, even in patients receiving concomitant therapy. Pembrolizumab requires further study to confirm a benefit for patients with refractory high grade glioma as monotherapy or in combination with chemotherapy or bevacizumab.

## Abbreviations

Bev: Bevacizumab
co-del: 1p19q codeleted
Con Bev: Concomitant bevacizumab
CR: Complete response
CTCAE: Common Terminology Criteria for Adverse Events
HGG: high grade gliomas
intact: 1p19q intact
KPS: Karnofsky performance score
methylated: MGMT methylated
ML: mutational load by MSK impact
MUT: IDH mutant
N: no
N/A: not applicable or unknown
OR: Objective response
ORR: Overall response rate
OS: Overall survival
PD: progressive disease
PD-1: Programmed cell death protein-1
PD-L1: Programmed cell death ligand-1
Pembro: pembrolizumab
PFS: Progression free survival
PR: Partial response
Prev Bev: previously progressed on bevacizumab treatment
Pt: Patient
SD: stable disease
unmethylated: MGMT unmethylated
VEGF: vascular endothelial growth factor
WT: IDH wild type
Y: yes
